# Evaluating Autonomous Urban Perception and Planning in a 1/10th Scale MiniCity

**DOI:** 10.3390/s22186793

**Published:** 2022-09-08

**Authors:** Noam Buckman, Alex Hansen, Sertac Karaman, Daniela Rus

**Affiliations:** 1 Computer Science & Artificial Intelligence Laboratory, Massachusetts Institute of Technology, Cambridge, MA 02139, USA; 2 Laboratory for Information & Decision Systems, Massachusetts Institute of Technology, Cambridge, MA 02139, USA

**Keywords:** mobile perception, robot platforms, autonomous vehicles, multi-robot systems

## Abstract

We present the MiniCity, a multi-vehicle evaluation platform for testing perception hardware and software for autonomous vehicles. The MiniCity is a 1/10th scale city consisting of realistic urban scenery, intersections, and multiple fully autonomous 1/10th scale vehicles with state-of-the-art sensors and algorithms. The MiniCity is used to evaluate and test perception algorithms both upstream and downstream in the autonomy stack, in urban driving scenarios such as occluded intersections and avoiding multiple vehicles. We demonstrate the MiniCity’s ability to evaluate different sensor and algorithm configurations for perception tasks such as object detection and localization. For both tasks, the MiniCity platform is used to evaluate the task itself (accuracy in estimating obstacle pose and ego pose in the map) as well as the downstream performance in collision avoidance and lane following, respectively.

## 1. Introduction

A major impediment to the adoption of autonomous vehicles (AVs) is the need to fully evaluate and test the fully autonomous vehicle hardware and software stack in realistic traffic scenarios. This is especially challenging for perception tasks, such as object detection and localization, which impact various components of the full AV stack and depend heavily on sensor configuration. Recent work [1,2] highlight the need for evaluating perception algorithms in the context of both the whole autonomous system and specific downstream tasks, such as obstacle avoidance, which is difficult to accomplish using existing datasets or simulators. In this work, we propose the MiniCity, a miniature autonomous vehicle platform for evaluating perception algorithms in a city-wide, multi-vehicle scale. In the MiniCity, 1/10th scale vehicles are equipped with full-scale hardware—Lidar, stereo cameras, and IMUs—and a full autonomy software stack—allowing researchers to evaluate their perception algorithm in isolation and the impact on the vehicle’s quality of autonomous driving.

Current tools for evaluating autonomous vehicle software and hardware consists of datasets, simulation, or full-scale vehicles. The high-cost and inherent safety risk of full-scale vehicles mean that most full-scale testing is limited to closed-course testing or tasks with limited interactions with other vehicles. Increasingly, researchers are relying on datasets or simulators for benchmarking the performance of their algorithms. Datasets [3,4,5,6,7,8] provide high-fidelity sensor recordings and are thus a popular choice for evaluating perception tasks such as object pose estimation and lane detection; however, they can not evaluate the impact on downstream modules such as trajectory planning or collision avoidance. Simulators [9,10,11,12] can allow for evaluating the full AV stack; however, they fall victim to the sensor sim-to-real gap and incur high computational costs for simulating multi-vehicle interactions. Miniature robot platforms [13,14,15,16,17] provide a middle ground in evaluation platforms, providing researchers with a lower-cost option that can enable real hardware testing while measuring an algorithm’s impact on both the individual task (object detection) and the impact on the rest of the autonomy stack (such as collision avoidance).

**Figure 1:**
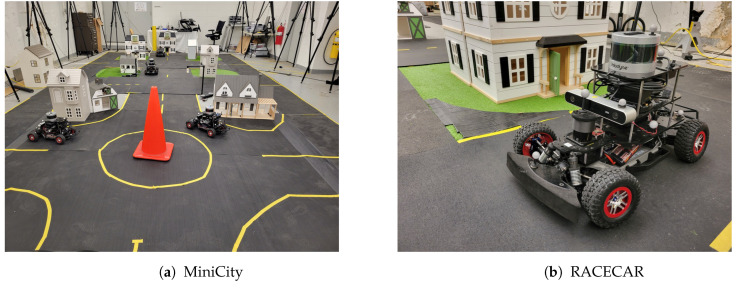
The MiniCity. (**a**) MiniCity consists of multiple intersections and vehicles for testing. (**b**) Miniature RACECARs equipped with sensors driving around scaled houses and grass.

The MiniCity (Figure 1) bridges the gap between real-world deployment and simulated testing. The MiniCity’s 1/10th scale urban setting consists of small-scale houses, roads, traffic lights, and fully autonomous vehicles, enabling researchers to test within a city setting without the dangers of real world testing. In this work, we highlight the MiniCity’s ability to evaluate a vehicle’s perception software and hardware, by deploying baseline perception tasks such as object detection and localization in interactive scenarios with multiple autonomous vehicles. However, other applications of the MiniCity include evaluating shared control between vehicles and human driver [18] and deploying novel intersection managers [19].

In summary, the main contributions of this paper are:

1. A small-scale multi-vehicle platform with scaled realistic assets, full implementation of AV stack and hardware;
2. A pipeline for evaluating the upstream and downstream performance of perception algorithms onboard miniature RACECARS;
3. Demonstration of the MiniCity’s evaluation capabilities for object detection and state estimation, using multiple hardware and software configurations

The remainder of the paper is organized as follows: in Section 2, we briefly summarize related works on testing platforms for autonomous vehicles and in Section 3, we describe the hardware and software platform for the MiniCity and RACECARS. Section 4 describes the software pipeline for evaluating the perception modules within the full autonomy stack, including metrics for evaluating the vehicle’s perception and localization stack. In Section 5, we report results in evaluating and comparing camera and Lidar-based object detectors and various sensor fusion configurations for localization.

## 2. Related Work

### 2.1. Simulation

Simulators such AirSim [9], Sim4CV [10] and CARLA [11], have become important platforms for evaluating complex systems such as autonomous vehicles, especially in areas where on-street deployment is limited. CARLA [11] is a popular vehicle simulation software that provides sensors simulation, road environments, and off-the-shelf planners for ado vehicles. Pylot [20] provides a testing suite on top of CARLA that can evaluate latency-accuracy performance at various points in the full AV pipeline. SUMO [21] simulates microscopic traffic at a city level, allowing for vehicle-to-vehicle communication, traffic demand modeling, and intersection management. VISTA [22] combines photorealistic simulation with full-scale vehicle logs to simulate and train AV neural networks. A main limitation in simulation is the gap between the realism of the simulation and the world, especially when it comes to simulating physical hardware, environments, and the behaviors of surrounding vehicles.

### 2.2. Datasets

For perception tasks such as object detection and behavior prediction, datasets such as the Kitti Dataset [3] have become the de facto standard for evaluating and benchmarking algorithm performance. More recently, autonomous vehicle companies have released datasets of sensor logs from full-scale vehicle fleets, such as NuScenes [4], Argoverse [5], Waymo Open [6], A2D2 [7], and Appoloscape [8]. These datasets have been proven quite effective in evaluating specific tasks such as localization, perception, and prediction, however, are not able to evaluate the performance of the full stack AV or its impact on downstream tasks such as planning and control. For example, while datasets can allow for comparing an object detector’s ability to detect a pedestrian, it can not measure whether one detector’s errors lead to significant safety risks compared to other detectors. In addition, the static environments (with non-responsive agents) do not capture complex interactions or the effect of the planning algorithm on the surrounding agents. This is especially important when developing planning algorithms in highly interactive scenarios such as at intersections where agents may react to other vehicles. Finally, datasets are limited to a fixed sensor configuration and road topology, whereas physical hardware platforms provide researchers additional flexibility.

### 2.3. Full-Scale Vehicle Platforms

The most comprehensive testing can occur with a full-scale, real autonomous vehicle platform. Full-scale research platforms include MCity [23] and individual vehicles such as Stanford’s Audi TTS [24], MIT’s Toyota Prius [25], UofT’s Zeus [26], while allowing for real sensors and varied environments, full-scale hardware is typically prohibitively expensive for most researchers, especially for scenarios requiring multiple autonomous vehicles. In addition, full-scale vehicles require heightened safety concerns, requiring most testing to occur on closed courses with limited interactions with other agents. In contrast, lower-cost research platforms can allow researchers to develop algorithms for the most dangerous scenarios and test autonomous vehicles at their limit, without concern for the safety of the researcher or the expense of the cars. Finally, these factors increase linearly with the number of vehicles, limiting researchers’ ability to test multiple autonomous vehicles simultaneously.

### 2.4. Small-Scale Vehicle Platforms

The MiniCity’s vehicles are based on MIT’s miniature racing platform, RACECAR [17] which was initially used for educational settings. These 1/10th scale vehicles are based on the Traxxas 1/10th Rally remote-controlled cars with Nvidia Jetson computers and additional cameras and Lidar sensors. Other scaled racing platforms such as Penn’s F1Tenth [13], Amazon’s DeepRacer [14], Georgia Tech’s AutoRally [15], have focused primarily on planning and control for racing. These racing platforms, such as AutoRally and DeepRacer, have been used extensively for deploying high-performance controllers using methods ranging from detailed vehicle dynamics modeling [15] to game-theoretic model predictive control [27] to reinforcement learning [14] and imitation learning [28]. These racing-based systems are usually limited to two vehicles on single loop race tracks, with high-speed control being the primary focus of these platforms. The MiniCity differs in its focus on evaluating the higher-level perception and planning, by providing city-like infrastructure, scenery, and interactive scenarios as well as a full AV software deployment, whereas scaled racing platforms focus on vehicle dynamics and control.

Most similar to the MiniCity, Duckietown [16] is an educational, open-source platform based on low-cost computing and sensing. The cheapest of the platforms, Duckietown relies only on computer vision for planning, limiting its ability to fully replicate the hardware and algorithms on modern AVs. In contrast, the MiniCity’s RACECARs have all the sensors found on a full-scale vehicle, from Velodyne Lidar to pseudo-GPS produced from our external motion capture. In addition, the MiniCity provides evaluation metrics and tools for monitoring the performance of each individual vehicle, with a focus on evaluating the upstream and downstream performance of the perception tasks.

## 3. MiniCity Platform Description

### 3.1. Physical Layout

**Figure 2:**
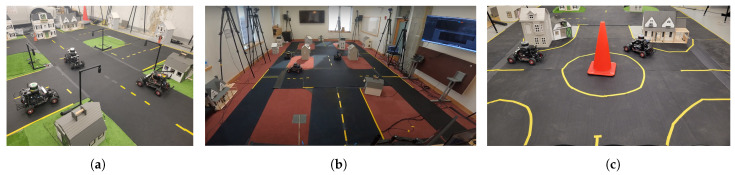
Three deployments of the MiniCity at MIT. (**a**) Small MiniCity; (**b**) Large MiniCity; (**c**) Roundabout.

The MiniCity is a 1/10th scale evaluation platform consisting of scaled houses, roads, and traffic infrastructure, multiple intersections for interactive scenarios, and external motion capture for evaluating the vehicle performance. The MiniCity’s roads are made from durable 2ft wide × 1/4${}^{\prime \prime }$ thick rubber gym mats with gaffer tape used for lane lines. Doll houses and synthetic grass are placed along the road to add realistic scenery and occlusions. The MiniCity’s photorealism allows us to deploy perception algorithms in environments that appear similar to deployment. The overall size of the MiniCity can expand to multiple intersections, with an overall length of 40 ft, or as short as 16 ft with a single intersection. Figure 2 shows three such configurations in different locations on MIT’s campus. The small size of the MiniCity relative to a full-scale city allows for experiments with various topological and environmental changes. All physical assets can be re-arranged for different road structures, weather settings, and scenery types.

### 3.2. Ground Truth Position and GPS-Spoofing from Motion Capture

**Figure 3:**
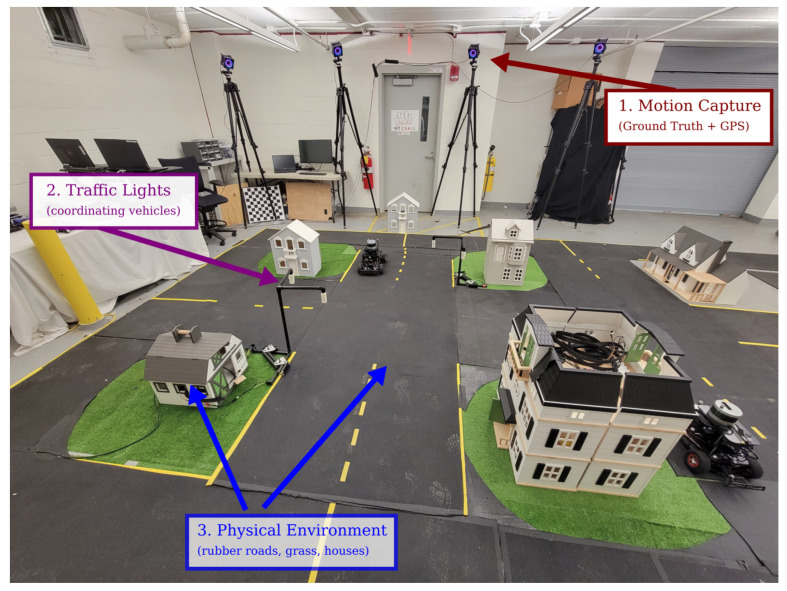
MiniCity Infrastructure. (1) External motion capture, (2) traffic lights, (3) physical roads and houses, and city maps allow multiple vehicles to drive around while testing a vehicle’s perception capabilities.

Ground truth localization is both necessary for evaluating localization and perception algorithms, and by providing simulated GPS signals to mimic outdoor environments. The MiniCity, as seen in Figure 3, includes a system of 10 Optitrack PrimeX 41 motion capture cameras deployed on portable tripods. The flexible setup allows for easily moving to new indoor and outdoor spaces. The Optitrack’s MOTIVE software tracks 8–12 passive reflective markers that are rigidly attached to each RACECAR, and publishes pose and orientation at 120 Hz.

Additionally, the motion capture system publishes a spoofed GPS signal to mimic GPS signals found in the real world using Robot Operating System’s (ROS) standard NavSatFix GPS message type. The GPS-spoofing module ingests the millimeter precise pose estimate of the vehicles and publishes a noisy position measurement with various types of noise, such as Gaussian, white, and brown noise distributions. The publishing rate of the GPS is reduced to a scaled rate of 5 Hz. The GPS signal characteristics can vary to better match realistic GPS scenarios, such as dropout due to signal attenuation, spatial variability found near buildings or in tunnels, sensor update frequency, and variable NMEA sentence information. The spoofed GPS sensors allow the MiniCity’s cars to use state-of-the-art state estimation and simultaneous localization and mapping (SLAM) packages without indoor modification, and provides for a fair comparison to outdoor testing when evaluating localization algorithms and sensor configuration, as we will describe in Section 5.

### 3.3. Mapping the MiniCity

**Figure 4:**
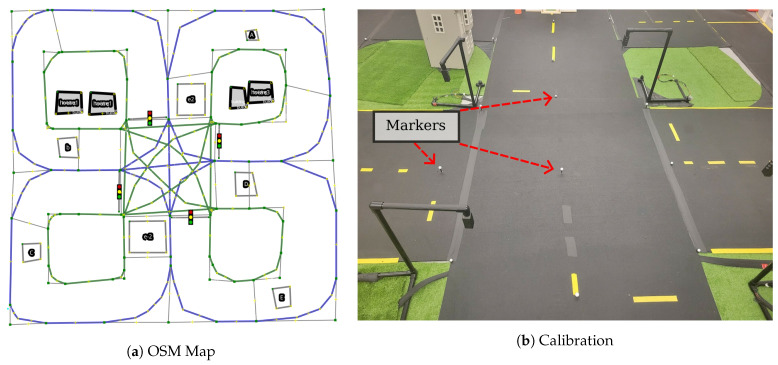
(**a**) OpenStreetMap map of the a single intersection with Lanelet labeling of the left (blue) and right boundaries (green). (**b**) Optitrack markers placed on roads give high quality ground truth position for map. The MiniCity map is used in autonomous navigating of the MiniCity and for evaluating downstream tasks such as traffic violations due to poor localization.

We provide maps of the MiniCity that are used for evaluating vehicle performance, lane line violations and traffic rules, and for use by the vehicle’s onboard planner. One advantage of the MiniCity’s scale is that high-definition mapping is less burdensome than real-world high-definition mapping of a full city. We map the 2D road geometry, lane lines, and building outlines in the OpenStreetMap (OSM) format, a popular open-source map format used for full-scale autonomous vehicle applications. The Lanelet2 API [29] provides semantic labeling for each road segment with information such as road direction, lane lines, traffic regulatory elements (traffic lights, speed limits), and overall road route structure. Figure 4a shows an example OSM with Lanelet2 map of the MiniCity with semantic information such as lane lines, virtual tracks, and traffic lights. The Lanelet2 API also builds a routing tree of the map’s road segments which is used by the car’s planner to navigate around the MiniCity.

Mapping of the road geometry begins with a rough manual outlining of the road structure of the MiniCity in JOSM, an OSM editor. We then place Optitrack reflective markers on the MiniCity surface (Figure 4b) corresponding to a subset of points on the initial OSM map. Correspondence between the Optitrack locations and the OSM map can be done manually, or automatically using an Iterative Closest Point algorithm for finding transformations between the Optitrack markers and nodes in the OSM. Each road segment and lane is defined within the Lanelet2 framework by providing the left and right road segment boundaries, which are then used by Lanelet2 to infer the direction of the road and generate a routing tree for navigating through the MiniCity.

### 3.4. Scaled Traffic Lights and Houses

**Figure 5:**
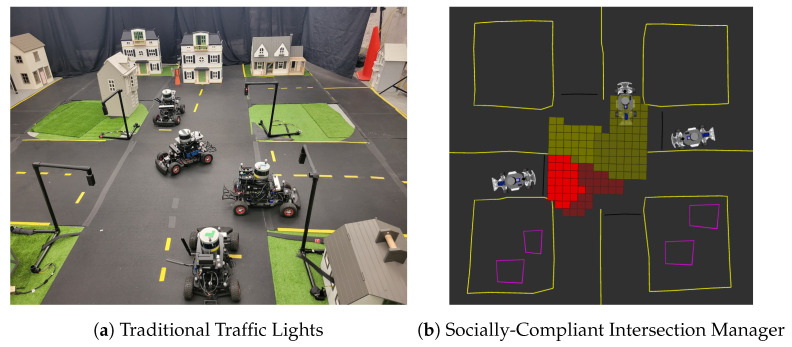
Traffic lights. (**a**) Multiple vehicles traverse intersections autonomously. (**b**) Traffic lights can operate in different modes, including intelligent socially-compliant reservation-based intersection managers.

Intersections and traffic signalling are unique features of city-wide driving. The MiniCity consists of multiple four-way intersections and roundabouts, which enables testing of perception algorithms in realistic traffic scenery and in complex scenarios such as a vehicle taking an unprotected left turn around occluded vehicles. The physical traffic lights (Figure 5a) consist of a to-scale plastic pipe structure, 3D printed enclosures, and pre-fabricated red-yellow-green LED board. A Raspberry Pi 4 controls the LEDs and communicates with the rest of the MiniCity software stack via ROS. The traffic lights can operate as traditional unsignalized (flashing red) and signalized (red-yellow-green) lights, or as intelligent traffic lights, such the socially-compliant autonomous intersection manager [19] shown deployed in the MiniCity in Figure 5b.

**Figure 6:**
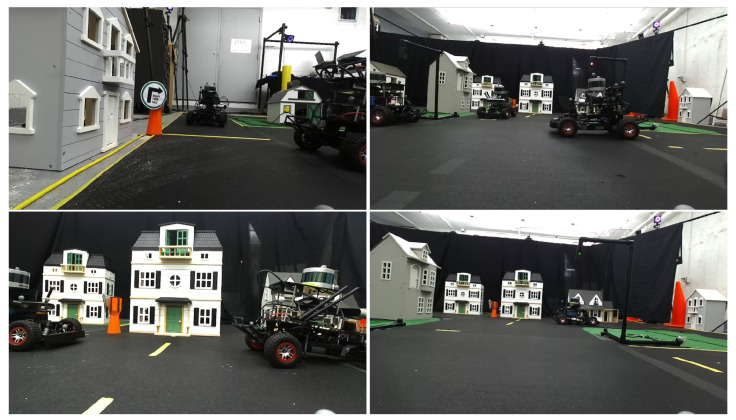
Views from the RACECAR driving in the MiniCity.

The MiniCity also consists of fake grass and doll houses to mimic background scenery during city driving. The houses also provide challenging perception scenarios such as occluded vehicles and obstructed pseudo-GPS. Figure 6 shows a few example views from the RACECAR’s onboard cameras that show how the MiniCity mimics full-scale driving scenes.

### 3.5. RACECAR Hardware

**Figure 7:**
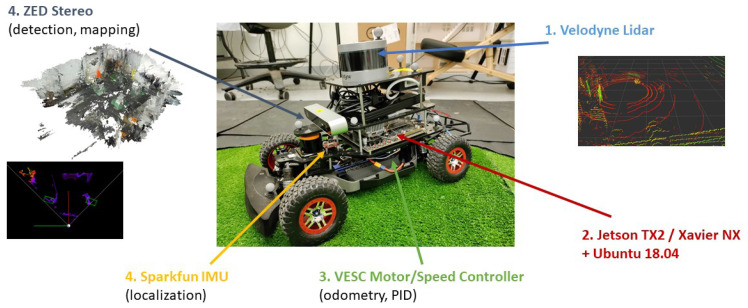
RACECAR Hardware Platform.

The MiniCity consists of 1/10th scale autonomous vehicles based on the RACECAR [17] platform, as shown in Figure 7. We provide configurations for multiple types of sensors and compute which allow for comparing various hardware configurations. The cars dimensions, including all equipment, are 0.53 × 0.30 × 0.33 m (L × W × H). They weigh 5.4 kg and have a turning radius of 1.4 m on the surface of our roads. For computing, the RACECARs use either an Nvidia Jetson TX2 or the newer Jetson Xavier NX, the latter consisting of an NVIDIA Volta GPU, 6-core ARM CPU, and 8GB RAM. The sensor suite is composed of a VLP-16 Velodyne Lidar, a Hokoyu 2d Lidar, a 9DoF Sparkfun IMU, a ZED stereo camera, and an Enertion FOCBOX speed controller that supplies wheel encoder odometry. The enhanced computation and sensing from previous versions of RACECAR and other educational platforms means we can deploy full-scale algorithms on the miniature vehicles.

**Figure 8:**
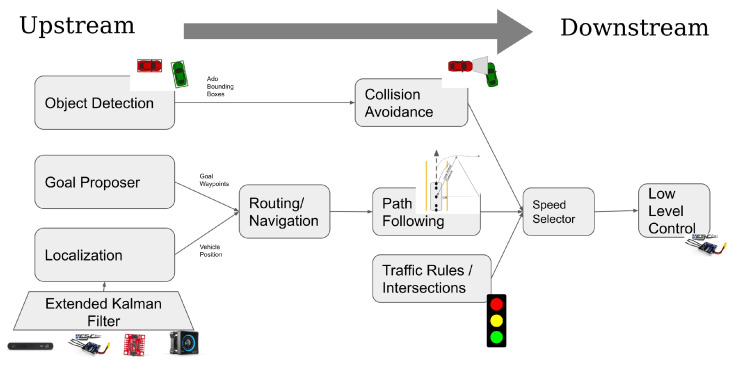
RACECAR AV Software Stack.

The platform’s code uses ROS Melodic for interprocess communication and external vehicle-to-all (V2X) communication. We compartmentalize each component of the pipeline into its own ROS Node to easily allow swapping algorithms and comparing component performance. Figure 8 shows the pipeline from upstream tasks (localization, perception) to low-level steering and speed control. For GPU intensive processes, such as object detection and lane detection, we implement the algorithm in NVIDIA’s Linux4Tegra Docker container and publish ROS topics over the host vehicle’s networking.

**Figure 9:**
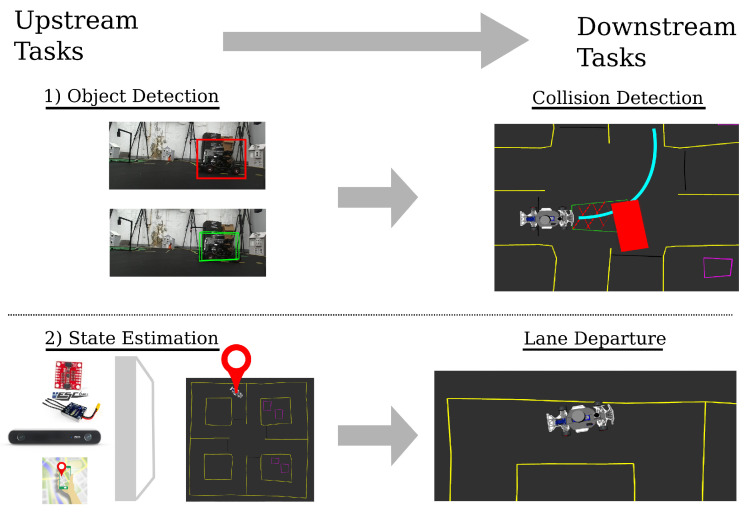
Upstream and Downstream Perception Tasks.

The low-level path follower implements a Pure Pursuit controller [30], during which a local goal point is selected at a fixed lookup distance ($l_{d}$) along the vehicle’s desired trajectory, and assumes a kinematic bicycle model to obtain a desired constant curvature path,

(1) $$\begin{array}{c} \gamma =\frac{2\epsilon_{l}}{l_{d}^{2}} \end{array}$$

where $\epsilon_{l}$ is the lateral distance between the vehicle heading and the goal position, γ is the computed curvature of the arc from the front wheel to goal position. The steering angle is computed using a proportional controller based on the curvature (δ=kγ). We choose a lookup distance $l_{d}$ that is twice the axle length of the car $\left(l_{d}=0.6\;m\right)$. The vehicle’s speed is determined with a proportional controller attempting to achieve the desired max speed, $v_{desired}$. The vehicle deploys a reactive collision avoidance detector, which checks whether any obstacles are located within a collision cone of the vehicle’s front bumper. The collision trapezoid, shown in Figure 9, extends the same lookup-distance $\left(l_{d}\right)$ ahead of the vehicle’s front bumper and spans a maximum width of 0.4 m.

## 4. Evaluating Upstream and Downstream Perception Tasks

### 4.1. Upstream and Downstream Tasks

Perception tasks typically are located at the very earliest, or upstream, stages of any autonomous vehicle stack. For example, the vehicle’s ability to estimate its own location in a map, directly affects the vehicle’s ability to generate trajectories and control the vehicle on the road. Similarly, the output of an object detector—pose estimates and bounding boxes of ado vehicles—directly impact an autonomous vehicle’s ability to avoid obstacles and drive safely. A main contribution of the MiniCity is the ability to safely test both the upstream and downstream performance of perception algorithms. A task such as detecting another vehicle in an image should be evaluated for both its ability to estimate bounding box poses (upstream) and the effect on the overall safety of the car for tasks such as collision avoidance (downstream). Whereas upstream evaluation metrics—intersection-over-union (IoU) or mean average precision (mAP)—may equally score the ability to detect vehicles close by from those far away, a downstream evaluator will properly penalize detectors that lead to an increasing number of collisions. This removes the need to create hand-crafted heuristics to capture the downstream effects and rather, we can directly measure the desired outcome of the perception tasks. Likewise, for state estimation, datasets can measure the estimation error of a given sensor configuration; however, it cannot measure whether different sensors cause increased traffic violations or collisions.

In this work, we focus on two examples of perception tasks, object detection and state estimation. These two tasks represent critical perception tasks that impact driving quality and safety. Figure 9 shows the two tasks—object detection and state estimation/localization—in the context of the upstream and downstream evaluations that are performed in the MiniCity. For object detection, the upstream task is defined as detecting and estimating the 6 degree-of-freedom pose (position and orientation) and bounding boxes of any vehicles within the field-of-view of the ego vehicle. The downstream evaluation occurs at the collision avoidance module, where the object detector is evaluated by its ability to prevent collisions as the vehicle drives through a busy intersection. For our second task, a state estimation pipeline ingests multiple sensor streams to compute a highly accurate and high-frequency estimate of the ego vehicle’s pose in the world frame, and downstream, we look to the lane departures of the vehicle as it navigates the MiniCity to evaluate the optimal sensor configuration.

### 4.2. Object Detection

Each RACECAR is equipped with both a Velodyne VLP-16 Lidar and Zed stereo camera, allowing us to test multiple classes of perception algorithms. We deploy two state-of-the-art object detectors using one or both of these sensors. Stereo-RCNN [31] feeds a pair of stereo images to a regions with convolutional neural network (RCNN) to predict key points, regions of interest (ROI), and object classes and finally 3D bounding boxes for each vehicle. Given that Stereo-RCNN’s classifier is typically pre-trained on Imagenet or similar datasets that lack pictures of RACECARS, we re-train the network using images of RACECARS. We also deploy PIXOR [32] which first creates a birds-eye-view feature map to input into a convolutional neural network (CNN) that computes a pixel-level estimate of the object’s pose and orientation. Both detectors are containerized in a Docker container and interfaced with the rest of the AV stack via ROS. The Stereo-RCNN and PIXOR detectors run at 0.1 Hz and 5 Hz, respectively. We observe a significant reduction in inference speed when running StereoRCNN onboard the Xavier NXs, from 0.20 s on an Nvidia Volta V100 to roughly 10 sec on a Jetson Xavier NX. For intermediate pose estimates needed for collision avoidance, a constant speed motion estimator predicts future poses.

**Figure 10:**
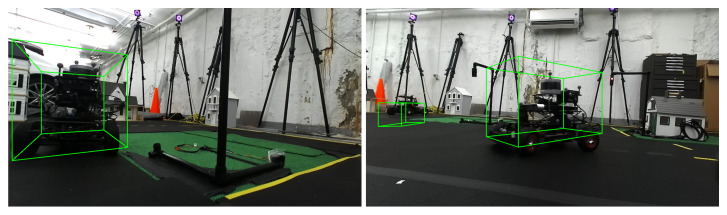
Ground truth bounding boxes (green) are automatically generated from the MiniCity’s external motion capture system. Ground truth bounding boxes can used for detector training, algorithm evaluation, and isolating downstream performance.

Each detector is re-trained using auto-generated training labels from the ground truth motion capture data of the vehicle pose, orientation, and dimensions (Figure 10). The ability to automatically label multiple vehicles with ground truth data from the external motion capture greatly streamlines and speeds up the training pipeline and prototyping cycle. In addition, the MiniCity can directly provide each vehicle with ground truth perception (ego and ado vehicle poses), allowing researchers to isolate and evaluate individual downstream components of the AV software stack such as planning and control. This helps in debugging so as to not propagate perception errors to lower down tasks.

### 4.3. State Estimation

State estimation is done through an Extended or Unscented Kalman Filter, implemented by the open-source robot_localization ROS package [33]. As inputs, the Kalman Filter takes odometry estimates from the onboard stereo ZED camera, the GPS from Optitrack, wheel encoder velocity estimates, and linear and angular accelerations from the Sparkfun IMU. The Unscented Kalman Filter (UKF) assumes non-linear process dynamics and noisy measurements ($z_{t}$) with non-linear noise model of the form

(2) $$\begin{array}{cc} \; & x_{t+1}=f\left(x_{t}\right)+w_{t} \end{array}$$

(3) $$\begin{array}{cc} \; & z_{t}=h\left(x_{t}\right)+v_{t} \end{array}$$

where $x_{t}=\left[x,y,z,\phi ,\psi ,\theta ,\dot{x},\dot{y},\dot{z},\dot{\phi }\ldots \ddot{\theta }\right]$, $w_{t}$ is the process noise with covariance *Q*, and $v_{t}$ is the measurement noise. The UKF uses an omni-directional, constant acceleration motion model so we assume a high process noise for $x,y,\theta ,\dot{\theta }$ to compensate for the model mismatch. We choose $Q_{x}=Q_{y}=Q_{\theta }=Q_{\dot{\theta }}=0.1m$ where $Q_{i}$ is the diagonal value of *Q* corresponding to state *i*.

The RACECAR’s initial pose estimate is updated by the motion capture and constantly updates its estimate as the vehicle drives in the MiniCity. The RACECAR uses the pose estimate to localize within the static OSM map, inferring its current lane for routing and its relationship to the intersection. In addition, the RACECAR’s onboard Zed stereo camera continuously generates a 3D map of the environment which can be used alongside the OSM for localization.

## 5. Perception Evaluation Results

In the following sections, we demonstrate the MiniCity’s ability to evaluate both upstream and downstream tasks for object detection (perception) and state estimation (localization). During evaluation, the OSM map and ground truth positioning provided by the Optitrack system become much of the backbone for the upstream and downstream evaluation metrics. Importantly, while an individual RACECAR does not necessarily have access to the Optitrack’s ground truth position or high definition map, the centralized performance monitoring uses this information to evaluate the algorithms onboard each vehicle in the MiniCity.

### 5.1. Evaluating Object Detection

The object detection evaluation begins with deploying each detector on the vehicle while running the full autonomy stack. An ado vehicle navigates the MiniCity autonomously as drone traffic, running its own collision avoidance and control. In addition, a human operator simulates high-risk scenarios such as an ado car speeding through an intersection or stopping at a crossroad. The ego vehicle operates autonomously with a mission of picking up and dropping drivers, using either a camera-based StereoRCNN detector or a PIXOR detector. As mentioned in Section 4, each detector is re-trained on a dataset captured in the MiniCity.

For a given detector, the MiniCity evaluates the upstream task of accurately detecting and estimating the pose of other RACECARs, by computing the intersection-over-union (IOU) of the 3D bounding boxes projected to the bird-eye-view plane, where intersection-over-union between predicted box $B_{pred}$ and ground truth box $B_{gt}$ is defined as

(4) $$IOU\left(B_{pred},B_{gt}\right)=\frac{area(B_{pred}\cap B_{gt})}{area(B_{pred}\cup B_{gt})}.$$

Since we expect multiple detections (ground truth and predicted), we first associate each predicted bounding box $B_{pred,i}$ with a ground truth bounding box by finding the ground truth box $B_{gt,i}^{match}$ with the maximum IOU

(5) $$B_{gt,i}^{match}=\arg\max_{j}IOU\left(B_{pred,i},B_{gt,j}\right).$$

We consider a detection to match a ground truth when $IOU\left(B_{pred,i},B_{gt,i}^{match}\right)>\alpha_{IOU}$ and we constrain a one-to-one matching between predicted and ground truth detections. The onboard detection recall and precision are presented in Table 1 in the first two columns with $\alpha_{IOU}=0.05$, while offline evaluation of detectors is utilized during training, online evaluation includes any environmental changes or hardware considerations. For downstream evaluation, we focus on the collision avoidance capabilities of the cars which is directly related to the accuracy of the object detector and pose estimator. We measure the number of human handovers per minute (due to collision errors) and the subsequent sensitivity (6) and specificity (7) of the collision avoidance detector. The sensitivity and specificity are defined using ground truth detections to evaluate the true positive and negative rate of the collision avoidance detector activating, and comparing with the actual activation of the collision avoidance (CA) module.

(6) $$\begin{array}{cc} \mathrm{sensitivity} & =\frac{\#\;\mathrm{True}\;\mathrm{Positive}\;\mathrm{CA}\;\mathrm{Activations}}{\#\;\mathrm{Ground}\;\mathrm{Truth}\;\mathrm{CA}\;\mathrm{Activations}} \end{array}$$

(7) $$\begin{array}{cc} \mathrm{specificity} & =\frac{\#\;\mathrm{True}\;\mathrm{CA}\;\mathrm{Deactivations}}{\#\;\mathrm{Ground}\;\mathrm{Truth}\;\mathrm{CA}\;\mathrm{Deactivations}} \end{array}$$

**Figure 11:**
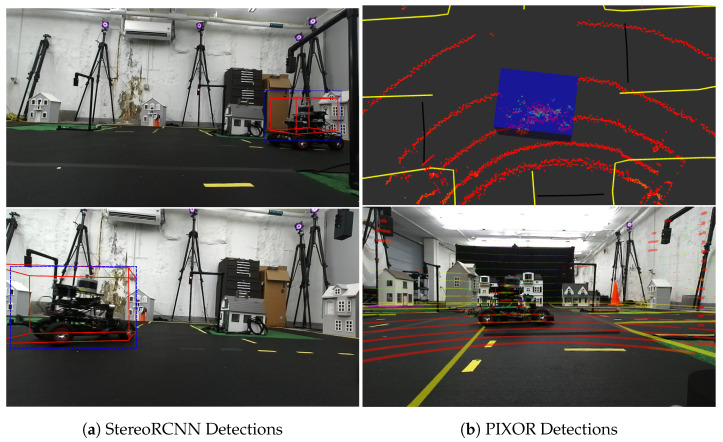
Example bounding box predictions from two different detectors. (**a**) StereoRCNN uses two stereo images to compute 3D (red) and 2D (blue) bounding boxes around other RACECARS. (**b**) PIXOR detector uses Velodyne point clouds as inputs to predict RACECARS 3D bounding boxes (blue).

In practice, both detectors are able to detect RACECARS in the MiniCity as shown in Figure 11. However, the Lidar-based PIXOR detector outperforms the camera-based detector both in upstream and downstream testing. PIXOR benefits from faster processing time due to the lower dimension input (due to PIXOR’s pre-processing) and leads to improved prediction for downstream collision avoidance. In contrast, StereoRCNN’s larger neural network, requiring two Resnet-101 for the region proposal network, leads to poorer performance in running realtime on the embedded GPU. In addition, the Lidar-based detector is robust to change in lighting conditions in the MiniCity.

### 5.2. State Estimation

For state estimation, we focus on the relative contribution of various sensor modalities on the overall quality of the onboard state estimation of the vehicle’s pose. Specifically, we evaluate the effect of each sensor on the vehicle’s estimate of its position $p={\left[x,y,z\right]}^{T}$ and orientation represented by quaternion q in the MiniCity’s reference frame. We use the high-quality ground truth pose provided by Optitrack to compare the state estimate to the ground truth pose, for various sensor configurations. Results for position error and angular error are reported in Table 2, with

(8) $$\mathrm{Position}\;\mathrm{Error}\;\mathrm{Metric}=||p-p_{gt}{\left|\right|}_{2}$$

and

(9) $$\mathrm{Angular}\;\mathrm{Error}\;\mathrm{Metric}\;=||\log\left(R\left(q\right)R{\left(q_{gt}\right)}^{T}\right)||.$$

where R(q) is the rotation matrix corresponding to rotation q, and $p_{gt}$ and $q_{gt}$ are the ground truth position and orientation, respectively. The angular error metric gives values in the range $\left[0,\pi \right)$ and provides a bi-invariant metric for the angular distance between 3D angles [34]. For the upstream evaluation, we re-run the Kalman Filter with different sensor configurations and measure the relative position and angular error as a percentage difference from our baseline with all sensors (Row 1). For example, we find that our pose estimation performs best without linear acceleration measurements from our IMU since the vehicle’s highly variable pitch and roll angles (and their estimate errors) lead to high noise on acceleration estimates.

The vehicle uses the estimate downstream to route through the MiniCity, generate trajectories within the lane, and ultimately provide steering and velocity controls to track lane centerlines. In Table 3, we evaluate the downstream effects of various sensor configurations by evaluating the percentage of time the vehicle crosses a traffic lane lines, where a lane line violation is defined as any part of the car crossing a road border or yellow line. In addition, to quantify the severity of the line violations, we report the average percentage of the car body that crosses over the line during a line violation. For downstream evaluation, we compare three localization configurations and repeat each run four times. We find that when utilizing the full sensor suite for localization (IMU, GPS, encoder), the lane violations correspond to only a very small percentage of the body over the line. In addition, not only does the quantity of line violations increase as we remove sensors (2× and 3× for GPS-only and IMU+Encoder-only), but also the severity of lane violations increased with a larger portion of the vehicle leaving the lane during a given lane line violation.

### 5.3. Limitations

A limitation of an experimental platform such as the MiniCity is that there remains a sim-to-real gap between the experimental setup and the full-scale autonomous vehicles. First, the MiniCity’s assets are not perfectly scaled copies of the full-scale vehicles and environment (e.g., dollhouses), meaning that most perception algorithms must be re-trained as we describe in Section 4. This limits the MiniCity’s abilities to evaluate out-of-the-box algorithms. Second, for non-perception applications such as designing high-performance controllers for collision evading maneuvers, a gap exists between the physical vehicle dynamics of the RACECAR’s and full-scale vehicle dynamics. For that reason, dynamics-specific tasks such as collision evading maneuvers may not transfer to full-scale as well as other tasks such as state estimation. Future work incorporating dynamics-mimicking controllers can help simulate dynamics similar to [35]. Finally, other hardware limitations, such as the RACECAR’s scaled onboard computers and power supply, can degrade the performance of power- or computer-intensive algorithms, and while power and computing are also issues for full-scale vehicles, scaled hardware can disproportionally affect algorithmic performance.

## 6. Conclusions

In this work, we present a novel platform for evaluating various hardware configurations and perception algorithms. The MiniCity enables closed-loop testing of the autonomy stack while evaluating the upstream and downstream performance of perception algorithms such as object detection and vehicle state estimation. Future work includes studying human driver behaviors around autonomous vehicles in the MiniCity, adding dynamic obstacles and simulated vehicle dynamics to improve collision simulations, and deploying and evaluating novel algorithms for safe autonomous driving. The MiniCity’s realistic scenery, baseline implementation of autonomous vehicle algorithms, and performance evaluation metrics enable researchers to fully explore the implications of new hardware and algorithms, including benchmarking against other algorithms and considering hardware limitations when deployed on vehicles. The MiniCity, as a tool for benchmarking and testing autonomous vehicles, is another important component in deploying safe full-scale autonomous vehicles onto city roads.

## Figures and Tables

**Table 1:** Evaluation of object detectors in the MiniCity.

Method	Detections		Handovers	Collision Avoidance	
	Recall	Precision	per min	Sensitivity	Specificity
Ground Truth	-	-	0.00	0.89	0.98
StereoRCNN [31]	0.061	0.091	2.05	0.16	0.93
PIXOR [32]	0.442	0.559	0.39	0.80	0.73

**Table 2:** Upstream evaluation of localization algorithms with different sensor configurations.

	Position Error			Angular Error		
Sensor	Mean	Stdv.	Change	Mean	Stdv.	Change
Configuration	(m)	(m)	(%)	(-)	(-)	(%)
All Sensors	0.1465	0.013	-	0.1458	0.016	-
No Zed/GPS	0.1757	0.029	19.94	0.1445	0.012	−0.91
No Zed	0.1465	0.013	−0.04	0.1445	0.012	−0.87
No GPS	0.2152	0.086	46.90	0.1445	0.015	−0.89
No IMU	0.1468	0.014	0.18	0.1513	0.021	3.74
No linear IMU	0.1464	0.013	−0.09	0.1484	0.018	1.78
IMU + Encoder Only	0.1742	0.027	18.86	0.1459	0.013	0.06

**Table 3:** Downstream evaluation of state estimation averaged over four runs for each state estimation configuration.

Sensor	Frequency of Line Violation	Severity of Line Violation
Configuration	(% of Run Duration)	(% of Car Body over Line)
GPS + IMU + Encoder	10.3	2.7
GPS-Only	23.3	6.3
IMU + Encoder Only	35.4	16.1

## Data Availability

The data presented in this study are available on request from the corresponding author.
